# Aging does not affect auditory motion discrimination based on interaural level differences

**DOI:** 10.1177/20416695241311206

**Published:** 2025-03-02

**Authors:** Shinya Harada, Ryo Teraoka, Naoki Kuroda, Wataru Teramoto

**Affiliations:** Faculty of Humanities and Social Sciences (Psychology), 13205 Kumamoto University, Kumamoto, Japan; Graduate School of Engineering, 13317 Muroran Institute of Technology, Muroran, Hokkaido, Japan; Faculty of Humanities and Social Sciences (Psychology), 13205 Kumamoto University, Kumamoto, Japan; Faculty of Humanities and Social Sciences (Psychology), 13205 Kumamoto University, Kumamoto, Japan

**Keywords:** aging, auditory motion perception, interaural level difference, motion processing

## Abstract

It is well known that aging affects fundamental perceptual functions. Numerous studies have investigated age-related changes in visual motion perception and demonstrated that aging impairs motion processing. However, limited studies have explored age-related changes in auditory motion perception, and whether aging influences auditory motion perception based on interaural level differences remains unknown. This study examined age-related differences in the discrimination of auditory motion direction based on interaural level differences. We conducted two experiments to estimate the signal-to-noise ratio and motion coherence thresholds required to discriminate auditory motion and visual motion directions, respectively, in younger and older adults. Results showed that age significantly impairs visual motion discrimination; however, it does not impair auditory motion discrimination. These findings suggest that aging does not affect auditory motion perception based on interaural level differences, at least with the broadband noise used in this experiment.

## How to cite this article

Harada, S., Teraoka, R., Kuroda, N., & Teramoto, W. (2025). Aging does not affect auditory motion discrimination based on interaural level differences. *i-Perception*, *16*(1), 1–9. https://doi.org/10.1177/20416695241311206

It is widely recognized that aging degrades perceptual and cognitive abilities. Previous research demonstrates that such degradation occurs in both higher-order functions (visual attention: Trick & Enns, 1998; short-term memory: Craik & McDowd, 1987) and fundamental perceptual functions (visual acuity: Milne, 1979; contrast sensitivity: Lord et al., 1991; hearing: Wiley et al., 1998).

Previous studies have suggested that aging also impairs visual motion perception (Billino & Pilz, 2019). In particular, it is well known that aging affects global motion perception (Arena et al., 2012; Hutchinson et al., 2014; Trick & Silverman., 1991). Researchers have generally used random dot kinematograms that move translationally in specific directions and asked participants to discriminate the motion direction of the visual stimulus. In these experiments, the motion coherence of the visual stimulus was varied to determine whether aging affected the motion coherence threshold (Supplemental Figure S1). In other words, participants were asked to discriminate the motion direction of the target dots among the dots that served as noise. Although it was based on motion speed and stimulus size (Hutchinson et al., 2014; Sepulveda et al., 2020), many studies reported that the coherence threshold was higher in older adults than in younger adults (Billino & Pilz, 2019, see for a review), suggesting that global motion perception was impaired by aging. These age-related changes in motion perception may be caused by age-related organizational changes in the eyes (for a review, Lin et al., 2016) and/or by age-related changes in activity in the brain regions responsible for motion processing, particularly in the V5/MT area related with global motion perception (Biehl et al., 2017).

While numerous studies have examined age-related changes in visual motion perception, few have addressed age-related changes in auditory motion perception (Ludwig et al., 2012; Saberi et al., 2022). Notably, to the best of our knowledge, only one study (Saberi et al., 2022) has examined auditory motion perception in older adults. Saberi et al. (2022) investigated whether age differences affected the interaural delay threshold to detect auditory motion. They presented sound stimuli that simulated auditory motion along the interaural axis, created by adding a linear phase shift to noise bursts across the two ears. The result showed that the threshold of auditory motion detection was not significantly different between younger and older adults, which suggested that aging did not affect auditory motion perception in their study. Saberi et al.'s study is important as the authors were the first to explore auditory motion perception in older adults. However, previous studies have suggested that stimulus characteristics affected age-related differences in visual motion perception (speed: Sepulveda et al., 2020; size: Hutchinson et al., 2014). Additionally, the older adult group in Saberi et al.'s study (2022) comprised only six and two individuals in their 60s and 70s, respectively, out of 15 members (age range 48–72 years). Furthermore, a previous study also suggested that the visual global motion process degraded after 70 years of age (Arena et al., 2012). Hence, concluding that aging does not affect auditory motion perception based solely on Saberi et al.'s (2022) findings is premature.

This study investigated age-related changes in auditory motion perception between younger adult (aged 20–21 years) and older adult group (aged 71–82 years). We used pink noise as an auditory target stimulus and gradually modulated the interaural level difference to induce an auditory translational motion perception (Supplemental Figure S2). Additionally, we introduced a different pink noise as a distractor and measured the signal-to-noise ratio threshold to discriminate the auditory motion direction. A previous study reported that aging impaired lateralization discrimination based on interaural level differences (Goupell, 2022). Therefore, we hypothesized that age differences would affect auditory motion perception based on interaural level differences. In addition, this study aimed to confirm whether age differences affect the coherence threshold to discriminate global motion in visual modality. Several psychophysical studies investigated audiovisual integration in motion perception and suggested that the auditory motion perception process influenced visual motion perception and vice versa (Hidaka et al., 2011; Kim et al., 2012; Soto-Faraco et al., 2002, 2004). A functional magnetic resonance imaging study revealed that the activity of the V5/MT area, which was associated with visual global motion processing, was also enhanced by auditory motion (Alink et al., 2008). Therefore, we expected that the results of the visual motion experiment would provide a reference for interpreting the results of auditory motion experiment.

## Method

### Participants

Twenty younger adults (five men, mean age ± *SD*: 20.7 ± 0.81 years) and 25 community-dwelling older adults (11 men, mean age ± *SD*: 75.8 ± 1.94 years) participated in two experiments. For detailed information on the participants, please refer to the Supplemental Materials.

Before recruitment, we conducted visual acuity assessments for the older participants. Visual acuities were evaluated at viewing distances of 0.4 m and 3.0 m with both eyes open via Landolt C charts. Average visual acuities (including correction) at 0.4 m and 3.0 m were 0.68 (*SD*: ± 0.30) and 0.64 (*SD*: ± 0.22), respectively. Furthermore, hearing thresholds were assessed at 500, 1000, 2000, and 4000 Hz by pure-tone audiometry for each ear via an audiometer (AA-77A; RION Co., Ltd., Japan). Subsequently, the average across ears and these frequencies (PTA4) was calculated. We recruited participants who had a PTA4 of < 40 dB (i.e., normal–mild hearing loss based on the criteria of the Japan Audiological Society, 2014). Additionally, a Mini-Mental State Examination was administered in which all older participants scored > 26. None of the participants reported having dementia, depression, eye diseases, stroke, or being currently treated with neuroleptics.

### Stimuli

In the auditory motion experiment, a pink noise burst employed as the target auditory stimulus (sampling rate: 44,100 Hz, duration: 1,000 ms), and it was presented via headphones. Amplitude difference between the right and left auditory stimuli was gradually modulated as a function of time to induce the perception of auditory motion. We set two conditions based on the amount of amplitude change: large and small. In the large condition, the amplitude variation was 100%, which resulted in a larger perceived distance of auditory motion compared with the small condition, where the amplitude was changed by 50%. Auditory motion direction (right or left) was randomly chosen in each condition. Additionally, each auditory stimulus comprised a target pink noise and another pink noise (uncorrelated with the target noise) as a distractor. We estimated the signal-to-noise threshold to discriminate auditory motion direction. Accordingly, we estimated the rate between target and distractor noise at which participants could accurately discriminate auditory motion direction. Before the auditory experiment, participants were instructed to adjust the sound to a comfortable level.

In the visual motion experiment, a random dot kinematogram served as visual stimuli. A total of 100 dots were presented at 11° × 11° in the center of the liquid crystal display, with each dot being 0.16° in size. The dots were light gray, and their luminance was 34.4 cd/m^2^. The direction of the dot motion, either rightward or leftward, was randomly determined for each trial. We set two conditions based on the velocity of dot motion: fast and slow. Velocity of the dot motion was 12.6°/s and 3°/s in the fast and slow conditions, respectively. Stimulus duration was 1,000 ms. The background was dark gray, and their luminance was 0.15 cd/m^2^. Motion coherence was manipulated to estimate the coherence threshold required to discriminate the dot motion. Therefore, we estimated the minimum percentage of dots that moved in the same direction at which participants could accurately discriminate the visual motion direction. For detailed information on the stimuli and experimental apparatus, please refer to the Supplemental Materials.

### Procedure

This study employed a three-down one-up staircase method to estimate a 79% threshold in both auditory and visual motion experiments. Participant's task was to answer the motion direction of the auditory or visual stimuli. The sequence of the two experiments was counterbalanced across participants. Within each experiment, four blocks of the staircase method were used; consequently, all participants completed eight blocks across both experiments. For more detailed information on the experimental procedure, please refer to the Supplemental Materials.

## Results

### Auditory Motion Discrimination Experiment

**Figure 1.**
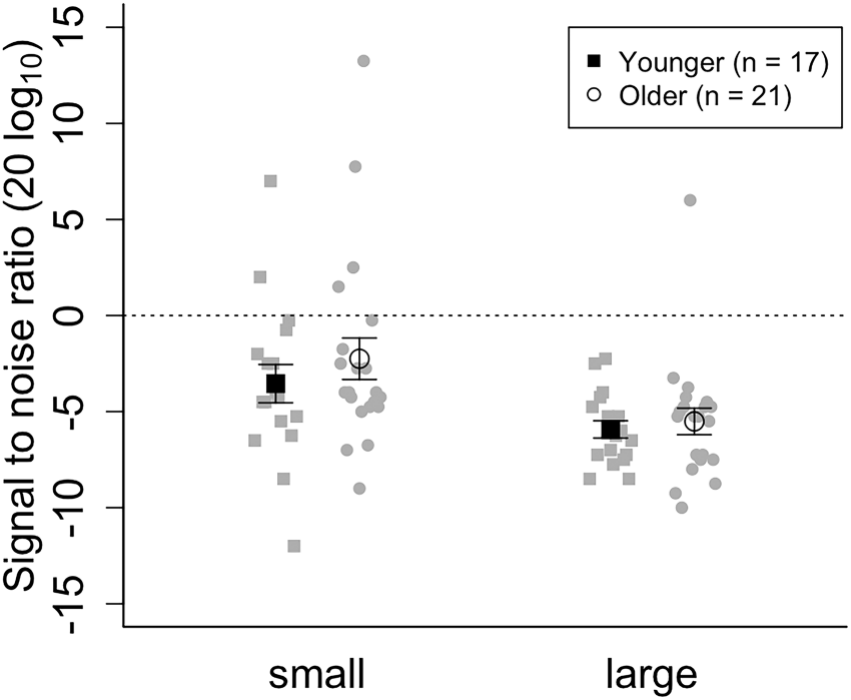
Auditory motion threshold. White circles and black squares represent the average threshold of older adults and younger adults, respectively. Gray symbols depict the data of each participant.

Statistical analyses were conducted using R software (4.2.1), anovakun (4.8.7) and JASP (0.18.1). Data from one older participant were excluded owing to a headphone disconnection during the auditory experiment. Additionally, we excluded the data from three younger and three older participants based on the criterion of each condition's average ± 2 *SD*. Consequently, the final analysis included 17 younger (*M* = 20.8, *SD* = 0.8) and 21 older participants (*M* = 75.5, *SD *= 1.8). Supplemental Table S1 presents the average hearing thresholds among the older adults. Hearing degradation was greater for high-frequency sounds than low-frequency sounds (Supplemental Table S1). Threshold of auditory motion discrimination was estimated by averaging signal-to-noise ratios from the last four reversal trials in the last two blocks. Figure 1 presents the average estimated signal-to-noise ratio threshold for discriminating the auditory motion direction. Shapiro–Wilk test for each condition did not confirm the normality of the data (*ps* > .05). Hence, we used the ARTool package in R and performed an aligned rank transform on the data (Elkin et al., 2021; Wobbrock et al., 2011). A two-way analysis of variance (ANOVA) for mixed design was conducted on the transformed data, which revealed a significant main effect of the variation condition (*F*(1, 36) = 30.16, *p* < .001, η_p_^2^ = .45). However, the main effects of age and interaction between variation and age were not significant (age: *F*(1, 36) = 0.25, *p* = .620, η_p_^2^ = .006; age × variation *F*(1, 36) = 0.24, *p* = .625, η_p_^2^ = .006). To assess the absence of significant differences between the age groups, we performed Bayesian *t*-tests and calculated the Bayes Factor (BF_01_) for each condition. In the small and large conditions, the Bayes Factor (BF_01_) was 3.035 and 2.237, respectively, indicating anecdotal evidence for the null hypothesis. For both younger and older participants, the average threshold in the small condition was higher than that in the large condition. Thus, contrary to our hypothesis, the signal-to-noise ratio threshold for discriminating auditory motion direction did not differ between the age groups.

We conducted exploratory Spearman's rank correlation analyses to examine the effect of individual differences in hearing thresholds. A previous study identified 1500 Hz as the boundary point of the interaural time and level differences (Moore, 2012); hence, we analyzed the correlations between the auditory motion threshold (slow or fast) and hearing average of all the frequencies as well as the subsets of frequencies (500 and 1,000 Hz, or 2,000 and 4,000 Hz). Additionally, we analyzed the correlations between the auditory motion threshold (slow or fast) and the average hearing threshold difference between the right and left ears. Corrections were applied via the Holm–Bonferroni method. Result revealed no significant correlation (slow vs. all frequencies: *p_adjusted_* = 1.0, rho = .12; slow vs. 500 and 1,000 Hz: *p_adjusted_* = 1.0, rho = −.07; slow vs. 2,000 and 4,000 Hz: *p_adjusted_* = 1.0, rho = .16; slow vs. difference between the right and left ears: *p_adjusted_* = 1.0, rho = −.27; fast vs. all frequencies: *p_adjusted_* = 1.0, rho = .08; fast vs. 500 and 1,000 Hz: *p_adjusted_* = 1.0, rho = .01; fast vs. 2,000 and 4,000 Hz: *p_adjusted_* = 1.0, rho = .05; fast vs. difference between the right and left ears: *p_adjusted_* = 1.0, rho = −.31).

### Visual Motion Discrimination Experiment

**Figure 2.**
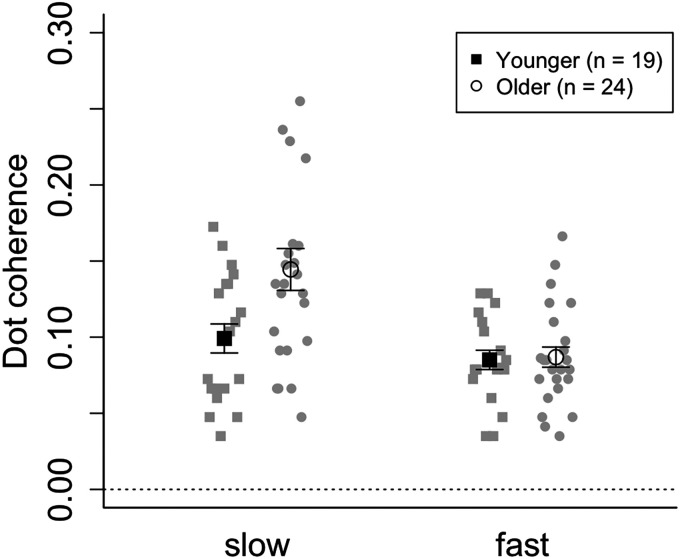
Visual motion threshold. White circles and black squares represent the average threshold of older adults and younger adults, respectively. Gray symbols depict the data of each participant.

Data from one younger and one older participant were excluded based on the criterion of the average of each condition ±2 *SD*. Therefore, 19 younger (*M* = 20.7, *SD* = 0.8) and 24 older participants (*M* = 75.7, *SD* = 2.0) were analyzed. Threshold of visual motion discrimination was estimated by averaging motion coherence from the last four reversal trials in the last two blocks. Figure 2 presents the average estimated motion coherence threshold for discriminating visual motion direction. Shapiro–Wilk test for each condition confirmed the normality of the data (*ps* < .05). A two-way ANOVA for mixed design revealed that the main effects of speed and age were significant (age: *F*(1, 41) = 15.94, *p* < .001, η_p_^2^ = .12; speed: *F*(1, 41) = 4.33, *p* = .043, η_p_^2^ = .044). Furthermore, the interaction between speed and age was significant (*F*(1, 41) = 5.88, *p* = .020, η_p_^2^ = .044). A simple main effect test revealed that the threshold difference between the age groups was significant in the slow condition (slow: *F*(1, 41) = 6.31, *p* = .016, η_p_^2^ = .13; fast: *F*(1, 41) = 0.04, *p* = .851, η_p_^2^ = .001). In addition, the difference between the slow and fast conditions was significant in older participants (younger: *F*(1, 18) = 1.95, *p* = .179, η_p_^2^ = .04; older: *F*(1, 23) = 17.37, *p* < .001, η_p_^2^ = .23). These results suggested that age difference could affect visual motion coherence threshold in the slow condition.

### Correlation Between Auditory and Visual Motion Discrimination Thresholds in Older Adults

Regarding the exploratory analyses, we conducted Spearman's rank correlation analyses between the auditory and visual motion thresholds in older adults. Since there were different final analysis participants between the visual and auditory experiments, we analyzed 20 older individuals in the correlation analyses. Correlation analysis was conducted for each pair (large vs. fast, small vs. slow, large vs. slow, small vs. fast), and the correction was made via the Holm–Bonferroni method. No correlations were significant (large vs. fast: *p_adjusted_* = .081, rho = .27; small vs. slow: *p_adjusted_* = 1.000, rho = .12; large vs. slow: *p_adjusted_* = 1.000, rho = .07; small vs. fast: *p_adjusted_* = .081, rho = .08).

## Discussion

We investigated whether age differences affected auditory motion perception based on interaural level differences. Our results revealed no age differences in the signal-to-noise ratio threshold in the auditory motion experiment, which was consistent with Saberi et al.'s (2022) finding, who found that age difference did not affect auditory motion perception. However, Saberi et al. (2022) varied the interaural delay of auditory stimuli and estimated interaural delay threshold to perceived auditory motion. To the best of our knowledge, this was the first study to suggest no age-related differences in auditory motion direction discrimination based on interaural level differences. However, why there were no age-related differences in auditory motion discrimination, unlike in the lateralization discrimination task (Goupell, 2022), remains unclear. According to classical duplex theory (Strutt, 1907), interaural time differences at lower frequencies and interaural level differences at higher frequencies are used for sound localization. Generally, age-related changes in hearing of low-frequency sounds were milder than those of high-frequency sounds (Ford et al., 2018). Therefore, Saberi et al. (2022) discussed that lack of a difference in the perception of auditory motion between age groups could be due to the low-frequency component of the auditory stimulus, which serves as a cue for auditory motion. Similar to previous studies (Ford et al., 2018), hearing degradation in older participants was greater for high-frequency sounds than low-frequency sounds in this study (Supplemental Table S1). However, the pink noise used in the experiment contained a sufficient amount of relatively low-frequency components. Thus, the effects of the low-frequency component as a cue to motion could explain the lack of age-related differences in auditory motion discrimination observed in this study. Additionally, Goupell (2022) utilized pure tone as the auditory stimulus, unlike our study that used a broadband noise. Therefore, age differences in auditory motion perception could be based on the stimulus characteristics. In this study, older participants had a higher visual motion coherence threshold than younger participants in the slow condition. This result was consistent with those of previous studies (Billino & Pilz, 2019, see for a review), which suggested that older participants in this study had reduced global visual motion processing compared with younger participants. Therefore, our results in auditory motion experiments were likely due to factors other than accidental bias, such as sampling older participants who had no degradation in visual motion processing. Another important point to consider is the individual differences in the hearing thresholds among older adults. We observed no significant correlations were found between auditory motion thresholds and individual differences in hearing threshold or threshold difference between the right and left ears. However, our small sample size may have influenced the results of the correlation analyses.

This study has some limitations. First, we targeted auditory motion perception induced by changes in the interaural level difference and estimated the signal-to-noise ratio threshold to discriminate auditory motion. In the context of visual motion perception research, various aging studies have utilized different participant tasks, stimulus forms, stimulus positions, and so on (Billino & Pilz, 2019, see for a review). Stimulus size and speeds affect age-related differences in motion discrimination, for example (Hutchinson et al., 2014; Sepulveda et al., 2020). Second, only two studies have examined age-related differences in auditory motion perception: Saberi et al. (2022) and this study. Therefore, we cannot definitively conclude that age-related differences do not affect auditory motion perception. Future studies should include further comprehensive experiments with varying stimulus characteristics. Third, we did not track the participants’ eye movements or include filler trials to confirm fixation. Consequently, we could not verify whether participants maintained fixation on the cross. However, in the visual motion experiment, global motion stimuli were presented at the center of the monitor. Therefore, we believe that the impact of fixation on the results was minimal in the visual motion experiment. Fourth, participants in our auditory and visual motion experiments differed slightly owing to outliers and trouble with headphones. These experiments differed in other aspects as well, such as stimulus speed and moving distance. Therefore, a further rigorous comparison of the auditory and visual motion results was not possible. Further research should strive for corresponding experimental designs between auditory and visual modalities to enable comparisons. Lastly, we were unable to detect the effects of individual differences in hearing thresholds owing to sample size. Further research should involve a larger sample size to better identify the effects of individual differences in hearing.

## Supplemental Material

sj-docx-1-ipe-10.1177_20416695241311206 - Supplemental material for Aging does not affect auditory motion discrimination based on interaural level differences Supplemental material, sj-docx-1-ipe-10.1177_20416695241311206 for Aging does not affect auditory motion discrimination based on interaural level differences by Shinya Harada, Ryo Teraoka, Naoki Kuroda and Wataru Teramoto in i-Perception
