# Supplementary material for: Aging does not affect auditory motion discrimination based on interaural level differences
Source: Iperception. 2025 Mar 2;16(2):20416695241311206. doi: 10.1177/20416695241311206 (PMC11874038; doi:10.1177/20416695241311206)
Supplement: sj-docx-1-ipe-10.1177_20416695241311206 - Supplemental material for Aging does not affect auditory motion discrimination based on interaural level differences [file sj-docx-1-ipe-10.1177_20416695241311206.docx]

**Aging does not affect auditory motion discrimination based on interaural level differences**

Shinya Harada, Ryo Teraoka, Naoki Kuroda, and Wataru Teramoto


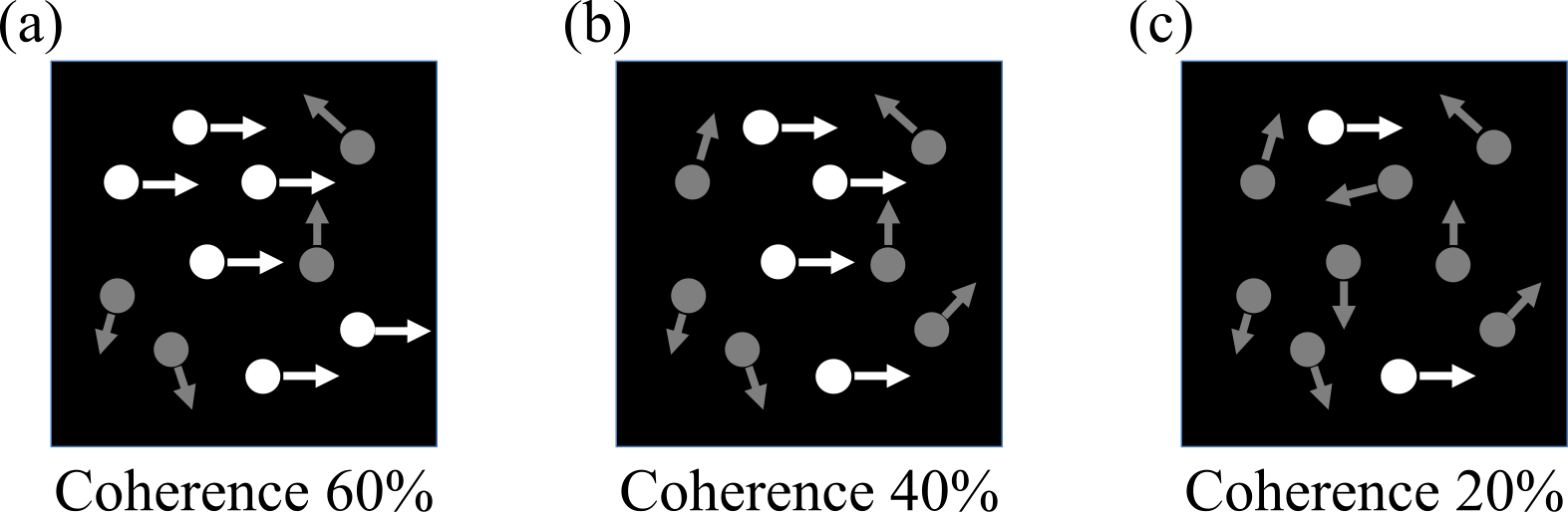


Figure S1. Schematic diagram of the global motion stimulus. Each motion coherence is (a) 60%, (b) 40%, and (c) 20%.


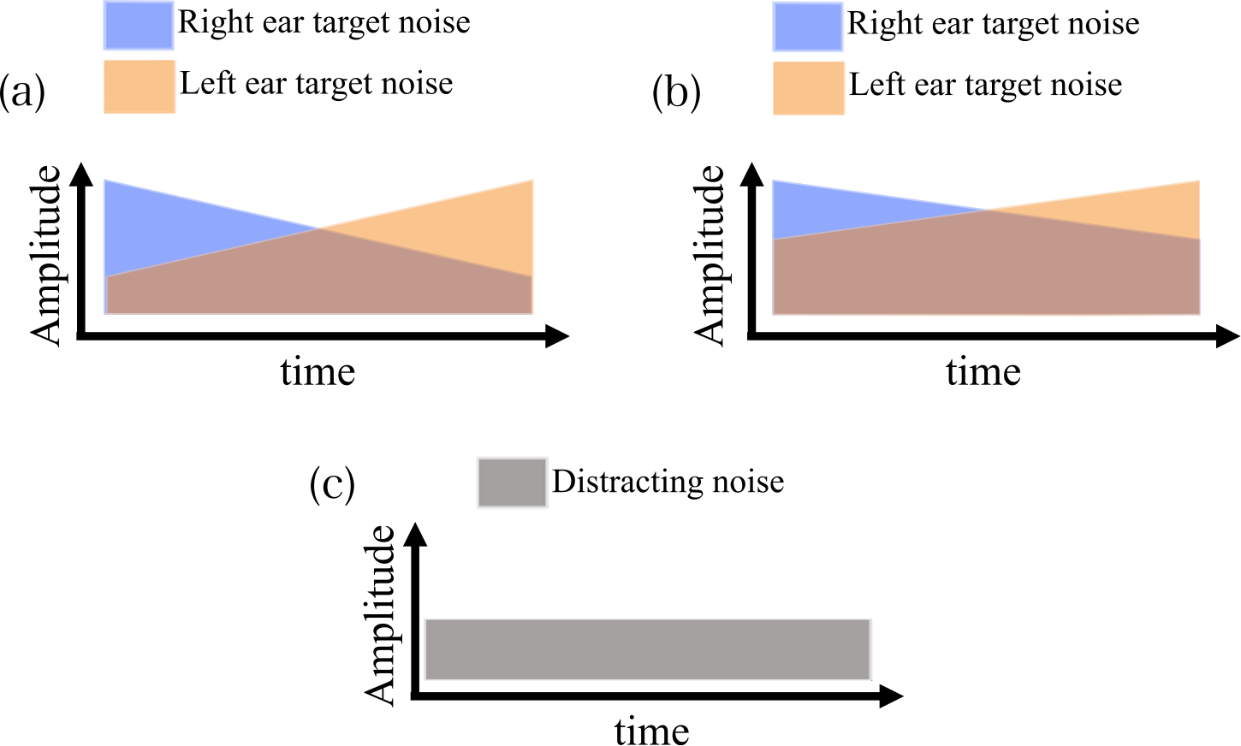


Figure S2. Amplitude of auditory stimuli as a function of stimulus duration. (a) Amplitude of target noise in the large condition, with blue and orange representing right ear and left ear noise, respectively. (b) Amplitude of target noise in the small condition, with blue and orange representing right ear and left ear noise, respectively. (c) Amplitude of distracting noise.

**Participants**

All the younger adults were undergraduate students at Kumamoto University. All older participants belonged to the Silver Human Resource Centers of Kumamoto, a temporary employment organization for older adults. We determined the sample size through a priori power analysis via PANGEA (Westfall, 2016). Analysis indicated that for a medium effect size (*d* = 0.40), a sample size of 20 participants was required in each age group (α error probability = .05, power [1-β error probability] = .80). We recruited 25 older participants because of greater variability in data among older individuals and the potential for data exclusion due to misunderstanding the instructions.

All participants were naïve to the experiment’s purpose. This study was approved by the Ethics Committee of the Kumamoto University. Furthermore, this study was conducted in accordance with the principles of the Declaration of Helsinki (1964). Written informed consent was obtained from all participants.

**Apparatus and stimuli**

Experiments were controlled and visual stimuli were presented using a Windows PC (Mouse Computer G-Tune P5-RT) and Liquid Crystal Display (EIZO FlexScan EV2480). Auditory stimuli were delivered via headphones (SONY MDR-CD900ST) through an audio interface (Roland QUAD-CAPTURE). An experimental program was developed via Python (3.10.9) and PsychoPy library (2022.2.5). Participant responses were recorded via a numeric keyboard.

To estimate the signal-to-noise ratio threshold, we created auditory motion stimuli based on the following equation:

$$\text{Signal-to-noise ratio }\left( \text{dB} \right)\text{ = 20 }\text{log}_{\text{10}} \frac{\text{target noise amplitude}}{\text{distractor noise amplitude}}$$

Target noise amplitude represented the initial value of the noise, which was gradually modulated as a function of time.

In each trial, a fixation cross appeared at the center of the display before the presentation, either as an auditory stimulus or random dot kinematogram. Participants were instructed to maintain their gaze on the fixation cross. The fixation cross was 0.6° × 0.6° in size, light gray in color, and had a luminance of 22.6 cd/m^2^.

**Procedure**

Termination criterion for the staircase method was reached when six reversals occurred. In the auditory motion experiments, the initial signal-to-noise ratio was set to 12 dB, with a step size of 8, 4, and 2 dB for the first, second, and subsequent four reversals, respectively. For the visual motion experiment, the initial visual motion coherence was set at 0.8, equivalent to 80% of dots moving in the same direction. Step size in the visual motion experiment was 20%, 10%, and 5% of dots for the first, second, and remaining four reversals, respectively.

Experiments were conducted in a dimmed, quiet room. Initially, participants were seated on a chair positioned 57 cm from the display, and their heads were fixed with a chin rest. At the beginning, the fixation cross was presented at the center of the display, and participants were asked to fixate on the cross as long as it was presented. The fixation duration was 1000 ms.

After the fixation cross disappeared, either auditory or visual stimuli were presented. In the auditory motion experiment, pink noise served as the stimuli, with only a background displayed on the monitor. In the visual motion experiment, a random dot kinematogram was presented, with no auditory stimuli. Duration of each stimulus was set to 1000 ms. After the stimulus disappeared, the fixation cross reappeared for 1000 ms, after which the response display appeared. Participants answered the motion direction of the auditory or visual stimuli via a key press. Upon pressing the response key, the display disappeared simultaneously, and the next trial began.

To sustain participants’ concentration, filler trials were integrated into both the experiments. In the auditory and visual experiments, filler trials featured signal-to-noise ratios of 11, 7, and 3 dB and motion coherences of 0.8, 0.7, and 0.6 to facilitate relatively straightforward discrimination of the auditory and visual motion directions, respectively. Filler trials were conducted randomly once in four times in both experiments.

Prior to the two main experiments, participants engaged in a practice session that comprised six trials. In practice, the signal-to-noise ratios and motion coherence mirrored those of the filler trials in both the experiments and were randomly chosen in each trial. Before the practice trials in the auditory motion experiment, participants were instructed to adjust the sound of the auditory stimuli to a comfortable level.

 Table S1.  Average hearing thresholds among older adults.


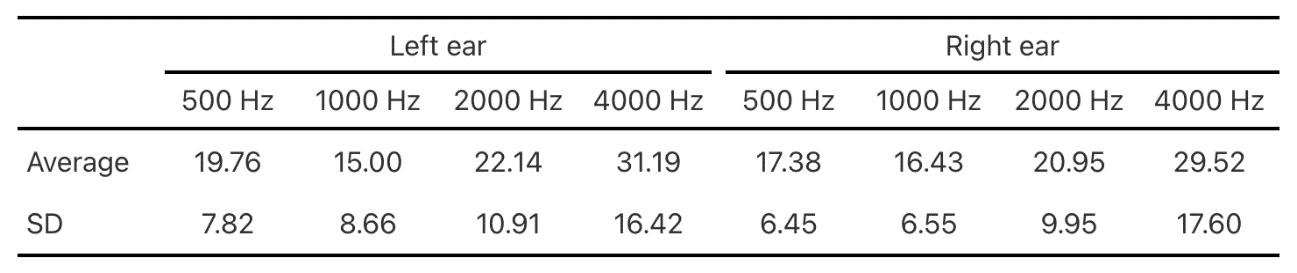


*Note.* Values indicate auditory thresholds (dB) for each frequency among the older adults.
